# Examining the Neighborhood Attributes of Recently Housed Partner Violence Survivors in Rapid Rehousing

**DOI:** 10.3390/ijerph18084177

**Published:** 2021-04-15

**Authors:** Charvonne N. Holliday, Kristin Bevilacqua, Karen Trister Grace, Langan Denhard, Arshdeep Kaur, Janice Miller, Michele R. Decker

**Affiliations:** 1 Department of Population, Family & Reproductive Health, Johns Hopkins Bloomberg School of Public Health, Johns Hopkins University, Baltimore, MD 21205, USA; kbevila1@jhmi.edu (K.B.); kgrace2@jhu.edu (K.T.G.); akaur8@alumni.jh.edu (A.K.); mdecker@jhu.edu (M.R.D.); 2 Hopkins Center for Health Disparities Solutions, Johns Hopkins Bloomberg School of Public Health, Johns Hopkins University, Baltimore, MD 21205, USA; 3 Department of Advanced Nursing Practice, School of Nursing & Health Studies, Georgetown University, Washington, DC 20057, USA; 4 Johns Hopkins Bloomberg School of Public Health, Johns Hopkins University, Baltimore, MD 21205, USA; ldenhar1@jhu.edu; 5 House of Ruth Maryland, Baltimore, MD 21218, USA; janice@hruthmd.org; 6 Center for Public Health & Human Rights, Johns Hopkins Bloomberg School of Public Health, Johns Hopkins University, Baltimore, MD 21205, USA

**Keywords:** intimate partner violence, rapid rehousing, housing instability, housing insecurity, homeless persons, neighborhood deprivation, residential segregation, social determinants of health, health consequences

## Abstract

Survivors’ considerations for re-housing following intimate partner violence (IPV) are understudied despite likely neighborhood-level influences on women’s safety. We assess housing priorities and predictors of re-housing location among recent IPV survivors (*n* = 54) in Rapid Re-housing (RRH) in the Baltimore-Washington Metropolitan Area. Choropleth maps depict residential location relative to census tract characteristics (neighborhood deprivation index (NDI) and residential segregation) derived from American Community Survey data (2013–2017). Linear regression measured associations between women’s individual, economic, and social factors and NDI and segregation. In-depth interviews (*n* = 16) contextualize quantitative findings. Overall, survivors re-housed in significantly more deprived and racially segregated census tracts within their respective regions. In adjusted models, trouble securing housing (B = 0.74, 95% CI: 0.13, 1.34), comfortability with proximity to loved ones (B = 0.75, 95% CI: 0.02, 1.48), and being unsure (vs unlikely) about IPV risk (B = −0.76, 95% CI: −1.39, −0.14) were significantly associated with NDI. Economic dependence on an abusive partner (B = −0.31, 95% CI: −0.56, −0.06) predicted re-housing in segregated census tracts; occasional stress about housing affordability (B = 0.39, 95% CI: 0.04, 0.75) predicted re-housing in less segregated census tracts. Qualitative results contextualize economic (affordability), safety, and social (familiarity) re-housing considerations and process impacts (inspection delays). Structural racism, including discriminatory housing practices, intersect with gender, exacerbating challenges among survivors of severe IPV. This mixed-methods study further highlights the significant economic tradeoffs for safety and stability, where the prioritization of safety may exacerbate economic devastation for IPV survivors. Findings will inform programmatic policies for RRH practices among survivors.

## 1. Introduction

Women who experience IPV are at greater risk of homelessness and housing instability for reasons strengthened by gender and race identity [1,2,3,4]. Women’s homelessness and housing instability may result from an immediate need to leave the violent situation [5,6], eviction due to financial abuse from their intimate partner or violence-related disturbances [7,8], and reduced employment and economic security [5,9,10,11]. Research estimates 60% of unstable IPV survivors seeking housing have children [4]. The heightened risk of violence among women with children [12], coupled with the impacts of IPV on housing stability, has important implications for maternal and child health. Entrenched racial and gender inequities reinforce the risk of housing instability and homelessness and the housing available to survivors, creating unique vulnerability profiles among Black women and other women of color throughout the life course.

**Figure 1:**
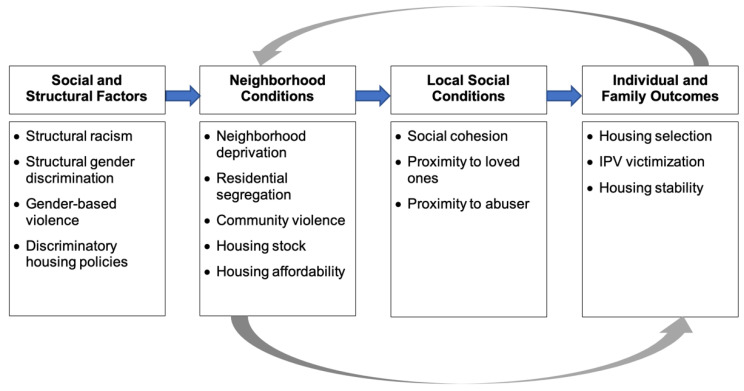
Hypothesized pathways among broad social and structural factors, environmental conditions, and housing outcomes among recent IPV survivors. Adapted from Massey, 1999 [15].

Housing is a social determinant of health [13]. The complex determinants of safety and housing selection result from structural racism and discrimination that impact neighborhood-level factors and social conditions (outlined in Figure 1). These broader factors contribute to “concentration effects [14],” where factors like deprivation and segregation reinforce racial and gender inequity [15]. Neighborhood deprivation and residential segregation, particularly among Black women [13,16,17], are associated with pre-pregnancy weight and gestational weight gain [18,19], hypertensive disorder during pregnancy [20], and perinatal mortality [21]. Residential segregation has also been associated with adverse birth outcomes among Black women, including preterm birth and low birth weight [17,22]. For women who survive IPV, such influences may increase their risk of violence, undermine social cohesion, and impact where they live [23,24].

The relationship between IPV and housing instability and homelessness has led to prioritizing housing services among IPV survivors at the federal level [6]. However, several barriers, including gender discrimination against survivors of IPV [25,26] and racism [25,27], may shape both the housing available to survivors and where they re-house. These barriers are critical in light of research that suggests neighborhood-level factors influence IPV risk [23,28,29]. Research indicates that these factors result from over a century of discriminatory housing policies in the United States that have promoted and reinforced residential segregation and pushed Black people and people of color into resource-deprived neighborhoods. At the neighborhood level, unemployment [30], low per capita and family income [30,31], low owner-occupancy [32], residential mobility [33,34,35], limited social cohesion [24], greater perceived neighborhood disorder [24,36,37], low collective efficacy [38], neighborhood disadvantage [33,39,40,41], high proportions of female-led households and families with children 18 years or younger [40], perceived and experienced community violence [42,43,44,45], and high alcohol outlet density are associated with increased risk of IPV [32]. Continued resource deprivation resulting from present-day structural racism in predominantly Black neighborhoods reinforces this risk while limiting the resources available to women experiencing violence [46].

Despite the established neighborhood-level risks for IPV and poor maternal and child health outcomes, there is a dearth of literature on how the housing available to survivors of IPV and where IPV survivors choose to live may impact their health, the health of their children, and their risk of future violence. Research suggests that systemic racism and deeply entrenched residential segregation have hindered housing interventions’ effectiveness in increasing access to high-quality housing in less-deprived neighborhoods [47]. However, obtaining housing in higher-opportunity neighborhoods may not necessarily correspond to increased subjective well-being [48].

Discriminatory housing practices intersect with race and gender, exacerbating challenges among survivors of severe IPV. This study examines the correlation between social determinants of health, including neighborhood attributes, and the housing location of recently housed IPV survivors participating in Rapid Rehousing (RRH), a federally funded, short-term rental assistance program. This mixed-methods study is the first to our knowledge to characterize neighborhood selection among recently housed IPV survivors. Findings from this research have implications on housing interventions and policies for IPV survivors.

## 2. Materials and Methods

### 2.1. Setting

The study occurred in Baltimore, Maryland, where residential segregation ordinances took root in 1910 [49]. Baltimore soon became a template for other cities’ adoption of discriminatory housing policies, resulting in formalized redlining policies nationwide [49,50]. Redlining is a form of structural racism initiated by the Home Owners’ Loan Corporation (HOLC) that was later reinforced by the federal government and real estate associations through formal and informal policies and practices. The HOLC outlined the boundaries of predominately Black neighborhoods in red to signify “at-risk” areas to be disqualified from investment and impeded Black families from purchasing homes in predominately White neighborhoods [51]. Restrictive covenants between property owners and real estate codes of ethics further segregated Black communities [50]. Such acts contributed to entrenched health disparities due to neighborhood disenfranchisement and a significant Black-White wealth gap [52] that stifled intergenerational wealth, which is often achieved through real estate [51,53].

In Baltimore City, the lingering impact of such policies is evidenced by the infamous “Black Butterfly” and “White L”, resemblances that demarcate clusters of deprivation and affluence [46]. The “Black Butterfly” outlines poverty, resource deserts, and a concentration of Black residents across the city’s East and West divisions. Conversely, the “White L” divides these two areas, extending into the Baltimore Inner Harbor and once-redlined surrounding areas marked by a significantly higher median income and life expectancy, an abundance of resources (e.g., electric scooters, well-resourced schools, free bus rides, a more significant number of business and mortgage loans), and a lower concentration of Black residents [46]. Recent evaluations and reports in lay media conclude that covert discriminatory practices are still effective today [50,54].

### 2.2. Participants

This study was completed with women who have experienced IPV and homelessness. For the sake of space, we refer to them as survivors because they were able to meet with us and share their experiences. Not all women who experience IPV are as fortunate. IPV survivors were recruited from a local domestic violence agency to participate in a longitudinal evaluation of two housing interventions—transitional housing (TH) and rapid re-housing (RRH). The RRH services were tailored to each survivor to include assistance in locating and obtaining housing and monthly rental assistance for a period determined by income level.

This analysis comprises baseline data from survivors in the RRH program (June 2019–December 2020). Individuals were eligible to participate in the study if they were at least 18 years old, identified as female, experienced past-year IPV, including physical or sexual violence or threats of violence, could participate in English, and were enrolled in RRH at the partnering site. Only survivors who were actively participating in RRH services and receiving rental assistance were enrolled in the study. Thus, survivors waiting to be housed or no longer receiving rental assistance were ineligible to participate until the inclusion criteria were met. Survivors who completed the final follow-up survey at six months were asked to participate in a qualitative interview until saturation was met.

### 2.3. Data Collection Procedures

#### 2.3.1. Survey Data Collection

We actively recruited survivors during their scheduled visits to the domestic violence agency and passively through flyers distributed by their service coordinators. Upon confirmation of eligibility, oral informed consent and baseline data collection took place in a private area within the agency. Housing program staff facilitated and accompanied our team for at-home informed consent and data collection for women who could not travel to the agency and to mitigate participant burden. All research activities were conducted remotely with the program and study staff’s assistance following state-wide restrictions on in-person research due to the COVID-19 pandemic.

Survivors completed a computer-assisted baseline survey via REDCap (Research Electronic Data Capture) [55,56], a secure, web-based data application. REDCap increased survivors’ privacy, confidentiality, and safety for the inclusion of geo-linked data. 

#### 2.3.2. Participant-Facilitated Geocoding

The feasibility of participant-facilitated geocoding was assessed to increase survivor safety, eliminating the need to collect survivors’ addresses. Instead, survivors self-recorded their census tract data using the US Census Bureau’s Geocoder [57]. Specifically, survivors were directed to the Geocoder website [57] through REDCap. They were instructed to input their current address and press “enter” to execute geocoding. Survivors copied the output (“GEOID” and “TRACT”) in a designated area of the active survey with support from the research assistant before deleting the browser history on the encrypted device. Women enrolled in housing programs are potentially the most at-risk of severe IPV and intimate partner homicide among IPV survivors [12,58]. Participant-facilitated geocoding strengthens safety protocols among this vulnerable population.

#### 2.3.3. Qualitative Data Collection

After completing the six-month follow-up survey, survivors were invited to participate in an in-depth interview via a secure web platform due to withstanding pandemic restrictions. Sixteen in-depth interviews were conducted by trained research assistants using a semi-structured guide between March 2020 and February 2021. Interview questions covered topics such as the participant’s housing selection, the impact of housing on abuse experiences, economic stability, children, the effect of the COVID-19 pandemic on housing, abuse, ongoing needs, and continued barriers to safety and housing stability. Survivors gave consent for digital audio recording. Recordings were transcribed by a transcription service and checked for accuracy by research assistants. Any identifying information inadvertently revealed was removed during transcription to ensure confidentiality. Digital recordings and transcripts were stored on a secure, encrypted computer. Survivors who completed the interview received a $25 retail gift card to thank them for their time.

#### 2.3.4. Ethical Considerations

Survivors completed the baseline survey in approximately 45 min. Each received a $15-dollar gift card for their time and resources on health-related topics, including violence. Trained research assistants conducted all of the data collection procedures after completing a comprehensive four-part training series. The Institutional Review Board of Johns Hopkins Bloomberg School of Public Health approved all methods, which were aligned with ethical best practices for violence-related research [59].

### 2.4. Measures

#### 2.4.1. Census Tract-Level Outcome Variables: Neighborhood Deprivation and Residential Segregation

Census tract-level outcome variables were constructed by linking survivors’ geocoded data with five-year census tract estimates (2013–2017) from the American Community Survey (ACS) [60] for four regions in the Baltimore-Washington Metropolitan Area: Baltimore City, Baltimore County, Montgomery County, Prince George’s (PG) County.

The modified neighborhood deprivation index (NDI) was created in an all-area model comprised of data from the four areas of interest, rather than individually, to create a comparable measure across regions [61]. Principal component analysis (PCA) determined the set of variables that explained the greatest proportion of variance in the first component across all regions [61,62]. Variables considered for inclusion in the index were chosen conceptually, based on previous women’s and maternal and child health studies [5,9,10,11,61,63]. Census tracts served as neighborhood proxies. Neighborhood-level variables from the ACS described broader physical, social and structural neighborhood conditions. The percentage of professionals in management positions, homes with more than one person per bedroom, women-headed households, vacant homes, unemployment, and families in poverty, receiving cash assistance, and receiving food assistance were considered. The percentage of professionals in management positions and households with crowded housing were dropped due to factor loadings below 0.25 [61]. All other factors were retained, explaining 64% of the final PCA variance, with loadings ranging from 0.31 to 0.45. The index was standardized to a mean of 0 and a standard deviation of 1. These methods are explained in-depth elsewhere [61]. The reliability of the index is high, with a Cronbach’s alpha = 0.88. The NDI is used continuously and categorically in this analysis; increasing positive values signify an increase in deprivation intensity.

Residential segregation at the census tract level used the Index of Concentration at the Extremes (ICE) [15], measuring the concentration of Black and White residents in each census tract across the four regions of interest. The ICE formula allowed for a calculation of residential segregation or degrees of racial concentration at the census tract level rather than county-wide, unlike other residential segregation measures [64]. The resulting residential segregation indicator ranged from −1 (most deprived) to 1 (most privileged). Census tracts with White residents only were designated as the extreme with most privilege, whereas Black only census tracts were the most deprived. 

#### 2.4.2. Covariates

Categorical demographic characteristics include age, race/ethnicity, education, and income. Past month employment was dichotomized to capture responses as affirmative or negative. 

An adapted measure for economic dependence on an abusive partner measured ten yes/no questions about things the participant may have depended on an intimate partner for in the past three months, such as food, transportation, and money [65]. A positive response to any question was considered economic dependence. A four-level variable assessed survivors’ ability to meet their financial needs independently, with assistance, partially with assistance, and not at all. This measure was developed by our community research partner as part of their Measuring Success Framework [66]. 

Food stress or insecurity was assessed using an adapted measure, “In the past three months, how often would you say you were worried or stressed about having enough money to buy food for yourself and your children?” [67] Similarly, recent housing insecurity was measured by, “In the past three months, how often would you say you were worried or stressed about having enough money to pay your rent or mortgage?” [67]

Variables measuring trouble getting housing in the past three months and recent difficulty with a landlord measures were adapted from Rollins et al.’s Housing Instability Index [68] and assessed dichotomously.

Investigator-developed measures assessed comfortability with proximity to family and friends (“I live close to family, friends, or other loved ones”) and the abuser (“I am comfortable that I live far enough away from (partner’s initials)”), dichotomized to no/not sure and yes based on ordinal responses of 1—strongly disagree to 5—strongly agree. A binary measure for home safety (“I feel safe at home”) and children in common with the abuser were recorded.

Perception of personal IPV risk was assessed using, “What do you think the chances are that you will be pushed, shoved, or hit by a partner in the next three months?” (1—not at all likely to 5—very likely) and recategorized to likely, unsure, and not likely, an adaption from previous work in the field [69].

The Social Cohesion Scale [70] consisted of four items assessing agreement with statements like “People in my neighborhood can be trusted.” Responses ranged from 1 (strongly agree) to 5 (strongly disagree) and were scored by averaging all responses for survivors who had complete data on all items; recategorized as yes, no, neutral. Cronbach’s alpha = 0.89.

Recent physical and sexual IPV was measured with five items from the physical and sexual subscales of the Revised Conflict Tactics Scale [71], which asked about the frequency of behaviors such as pushed, shoved or slapped me and insisted on sex when I did not want to, from 0 (never) to 3 (5 or more times). A variable in the analysis captures recent IPV based on the report of partner violence one or more times in the past three months; Cronbach’s = 0.89. Additional measures of health symptoms, such as depression [72], PTSD [73], and past year access to health care if needed, were included.

### 2.5. Data Analysis

#### 2.5.1. Quantitative Data Analysis

We conducted a descriptive analysis of the survivors’ demographic, economic, and health characteristics, and their social and structural environments (Table 1). Survivors were excluded from the analysis if they were missing census tract data (*n* = 5). We used linear regression with robust standard errors, controlling for potential heteroskedasticity, to assess mean differences in Neighborhood Deprivation Index (NDI) and residential segregation by the survivors’ demographic and economic characteristics, key housing variables, and social/community context. Statistical significance was set at *p* < 0.05. Fully adjusted models for each outcome (neighborhood deprivation and residential segregation) were run, including correlated variables with a *p* < 0.10 at the bivariate level. The potential for multicollinearity was assessed; the variance inflation factor (VIF) was ≤2.06 for both models (Table 2, Table 3).

**Figure 2:**
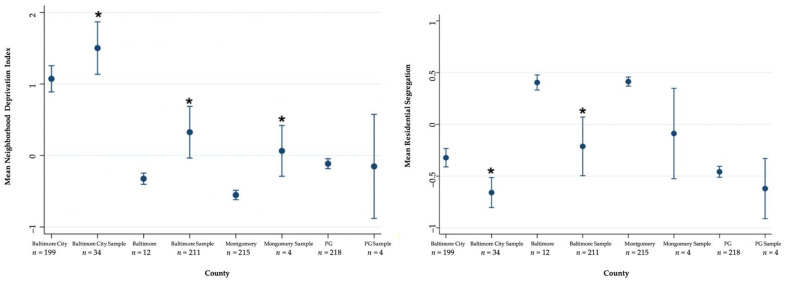
95% confidence intervals for mean neighborhood deprivation index (NDI) and residential segregation, comparing region-wide means and region-specific census tracts of the study sample (recently re-housed IPV survivors). N represents the number of census tracts in each location. * mean difference between the full region and study sample is statistically significant at *p* < 0.05.

**Figure 3:**
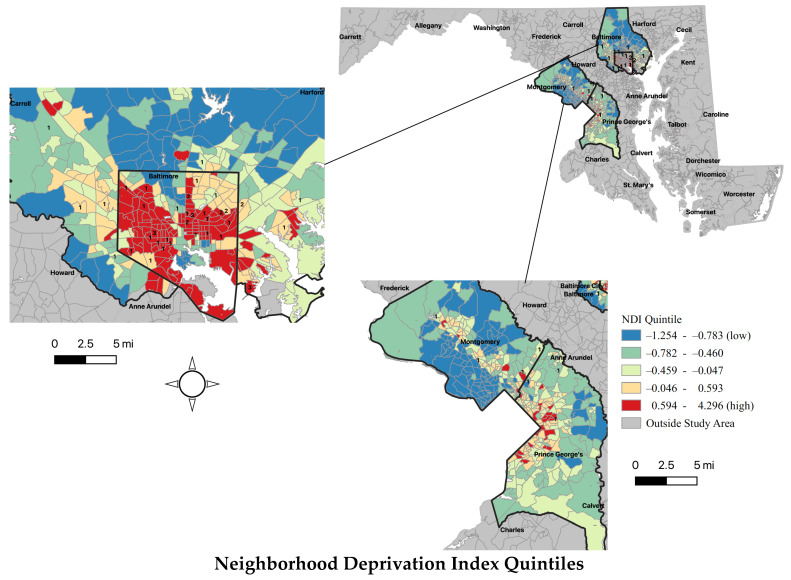

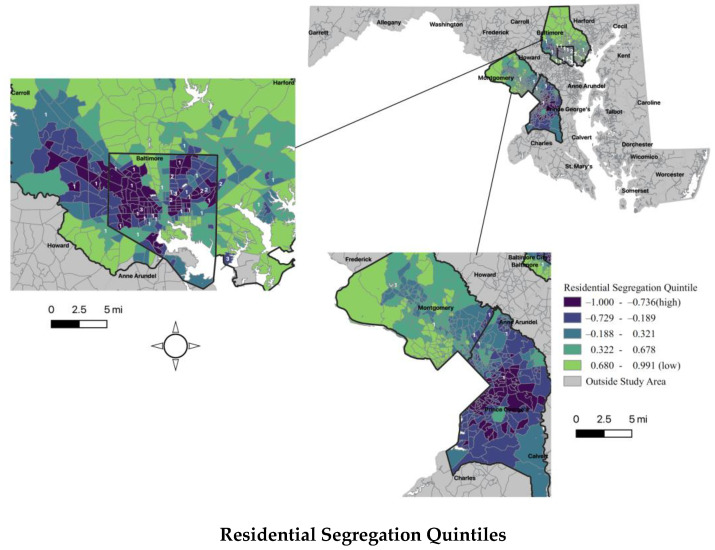
Spatial distribution of recently housed IPV survivors in Rapid Re-Housing by neighborhood deprivation and residential segregation quintiles. High residential segregation reflects a high concentration of Black residents relative to White, whereas low residential segregation reflects a high concentration of White residents relative to Black.

T-tests assessed mean NDI and residential segregation for the four regions of interest versus the study sample present in each of the respective geographic areas (Figure 2). These comparisons are displayed using forest plots. Finally, choropleth maps show the NDI and residential segregation quintiles across the regions and capture the distribution of study survivors (Figure 3). The maps were created using QGIS3.10 software [74], using the projected coordinate reference system (CRS) WGS 84/UTM Zone 18N. The county and census tract boundaries were downloaded from the Maryland state government’s publicly available data repository (MD iMAP) [75,76]. All statistical analyses were carried out in Stata/MP 15.1 [77].

#### 2.5.2. Qualitative Data Analysis

The research team created an initial codebook, which was iteratively refined throughout the analysis. Three authors independently applied codes to the first five transcripts and compared coding to resolve discrepancies and develop the codebook with emerging themes. The remaining transcripts were double coded by pairs of coders, who also compared coding to resolve discrepancies. Any unresolved issues or coding discrepancies were brought to the full study team for resolution. To enhance reliability, we conducted peer debriefing throughout the interviewing process, maintained an audit trail during analysis, and selected deviant cases, i.e., examples that contradict emerging patterns, post-analysis [78,79,80]. Saturation of data was reached after 16 interviews when no new codes or themes emerged.

Interview analysis followed the constant comparison method [81], whereby emergent themes are iteratively compared. The analysis focused on a subset of relevant deductive code families, including home selection, housing challenges, housing facilitators, neighborhood safety, and social networks. Code reports were aggregated, and relevant excerpts were extracted and summarized. Emergent themes were then organized around quantitative findings from this analysis. 

## 3. Results

### 3.1. Descriptive Statistics: Sample Characteristics and Neighborhood Indicators

The majority of the sample was Black (76%) and younger than 35 years old (63%) (Table 1). Most of the survivors had at least a high school diploma (92%). However, only a quarter earned more than $32,000 annually; about one-third of survivors lacked employment in the previous month. Only six percent of women in the sample were able to meet their financial needs independently, with the other portion meeting their needs with assistance (47%), partially with assistance (38%), or not at all (9%). Correspondingly, 40% of survivors were always worried or stressed about having enough money to buy nutritious meals in the past three months, and 57% were economically dependent on their abusive partner. Recent trouble securing housing (59%) and worry or stress about affording housing always or sometimes (87%) were also prevalent. A majority of women (78%) were living with post-traumatic stress disorder (PTSD) symptoms; more than half had experienced recent IPV. Within their neighborhoods, the survivors felt safe in their new home (87%) and were comfortable with their proximity to loved ones (58%) and the distance from their abuser (72%) (Table 1).

### 3.2. Spatial Distribution of Recently Re-Housed IPV Survivors in Study Areas and NDI and Residential Segregation Characteristics

The NDI among our sample ranged from −0.88 (least deprived) to 3.53 (most deprived). The mean residential segregation score is equal to −0.51 (SD: 0.48) for the census tracts (*n* = 43) in this sample, ranging from 0.66 (most privileged) to −1 (least privileged) (Figure 2).

Overall, survivors were re-housed in more deprived and more segregated census tracts when compared to the mean NDI and residential segregation of their respective regions (Figure 2). Survivors in Baltimore City (*n* = 34) lived in census tracts with the greatest deprivation (M = 1.50, SD = 1.09) and greatest level of residential segregation (M = −0.66, SD = 0.43) (Figure 2). Specifically, the mean NDI of survivors’ census tracts in Baltimore City, Baltimore County, and Montgomery County were significantly more deprived than the mean NDI of their respective regions. Mean NDI did not differ significantly between Prince George’s (PG) County and the census tracts with survivors in that region. Similarly, the mean residential segregation score was significantly different for survivors in Baltimore City and Baltimore County relative to the mean residential segregation in these respective areas; survivors lived in areas with a higher concentration of Black residents relative to White.

Choropleth maps depict the NDI and residential segregation quintiles (low to high) for census tracts within the four study areas (Figure 3). The spatial distribution of survivors by census tract is also presented in Figure 3, displaying the NDI and residential segregation of the census tract where they re-housed.

### 3.3. Factors Associated with NDI among Recently Housed IPV Survivors

In bivariate analyses, survivors with an annual income of more than $32,000 lived in significantly less deprived areas relative to those who earned $16,000 or less (B = −0.96, 95% CI: −1.61, −0.32; *p* < 0.05). Survivors who could meet their needs independently (B = 1.12, 95% CI: 0.20, 2.03; *p* < 0.05), with assistance (B = 0.94, 95% CI: 0.02, 1.86, *p* < 0.05) or only partially (B = 1.10, 95% CI: 0.15, 2.06, *p* < 0.05) lived in areas with greater deprivation than those who were unable to meet their needs at all. Trouble securing housing (B = 0.65, 95% CI: 0.04, 1.27, *p* < 0.05) also predicted increased neighborhood deprivation in preliminary bivariate analyses (Table 2).

In the final adjusted model, trouble securing housing in the past three months (B = 0.74, 95% CI: 0.13, 1.34; *p* < 0.05 and comfortability with the proximity to loved ones (B = 0.75, 95% CI: 0.02, 1.48; *p* < 0.05) were statistically significant predictors of NDI. Survivors unsure of their risk of abuse within the next three months re-housed in significantly less deprived census tracts relative to survivors who believed abuse in the short-term was unlikely (B = −0.76, 95% CI: −1.39, −0.14, *p* < 0.05). This trend is also observed among survivors who believed future abuse was likely (n.s.) (Table 2).

Other nonsignificant trends worth mentioning are economic dependence on the abusive partner and proximity to loved ones. In bivariate analyses, requiring financial assistance from an abusive partner was associated with re-housing in census tracts with significantly more deprivation when compared to economic independence from an abusive partner (B = 0.52, 95% CI: −0.06, 1.10; *p* < 0.10). Food, housing, and supplies for children/ childcare are what women depended on their partner for the most (data not shown). Comfortability with the proximity to loved ones (B = 0.58, 95% CI: −0.06, 1.22, *p* < 0.10) was also associated with increased neighborhood deprivation.

### 3.4. Factors Associated with Residential Segregation among Recently Housed IPV Survivors

In bivariate analyses, annual income between $24,001 and $32,00 per year (B = −0.37, 95% CI: −0.69, 0.04, *p* < 0.05) was associated with residential segregation, or living in a census tract with a greater concentration of Black people, relative to an annual income of $16,000 or less. The ability to meet needs independently (vs unable to meet financial needs) also predicted residence in a segregated area (B = −0.43, 95% CI: −0.83, −0.02, *p* < 0.05). In an adjusted model, having some high school education (vs some college) (B = −0.49, 95% CI: −0.88, −0.09) and economic dependence on an abusive partner (B = −0.31, 95% CI: −0.56, −0.06, *p* < 0.05) predicted re-housing in more segregated census tracts; occasional stress about housing affordability (B = 0.47, 95% CI: 0.14, 0.81, *p* < 0.05) predicted re-housing in less segregated census tracts (Table 3). Economic dependence (vs independence) on an abusive partner was marginally associated with residential segregation (B = −0.23, 95% CI: −0.50, 0.03, *p* < 0.10) in a bivariate analysis.

### 3.5. Considerations of IPV Survivor’s Re-Housing Decisions 

#### 3.5.1. Overview of Housing Location Considerations

Survivors prioritized housing locations that they could afford, where they could be safe from their abuser, have access to resources like their workplace and stores, and move into quickly. Fear of homelessness motivated the re-housing process.

Finding sustainable housing after the RRH program was a common theme, as one woman stated, “Everything’s pretty affordable for the most part. I was aware that once you get to the program after about six months, you’re on your own. I didn’t want to get anything I knew I couldn’t afford.” (1025)

Finding a home in a safe area away from or unknown to their abuser was also crucial. “I was trying to apply anywhere just to move from where I knew he knew I live, just trying to get away” (1032), and “I wanted to live outside the city. I knew that he was looking for me inside the city. He was actually using Baltimore City Police. I definitely did not want to stay in the city.” (1055)

Women prioritized the ability to move in quickly due to pressure to leave their current housing situation, which also impacted their re-housing location, “All I knew is the pressure was being put on me […] I just need to find a place soon” (1010), the need to escape their abuser, “To be honest, I didn’t even care. I just needed to get out. I was looking everywhere, so whatever came through, that’s what I jumped on” (1045), or out of desperation for a place to stay, “I took the first space open just so I wouldn’t be in the streets of Baltimore.” (1028)

Survivors’ rehousing process was further impacted by social and economic factors like their proximity to loved ones and housing challenges.

#### 3.5.2. Considerations of Family, Friends, and Other Loved Ones 

Women discussed their considerations of safety and social support. For one woman, safety outweighed being close to family and friends, and she was cautious about loved ones leaking her new address.

“I’m being really careful with my friends so that they don’t associate my name and my new address. Who knows how it gets leaked? But, at the same time, it’s still not too far away for them to visit me. It’s much more [important] for me and the children to live in a safe home away from friends than living in a place where I have to hide more, where I have to worry about if he is looking for me.”

(1055)

Women with family in other states, whom they could not rely on for immediate shelter, were concerned about finding housing. They prioritized staying in a familiar neighborhood with familiar neighbors to call on for help.

“I told my friends and family in North Carolina (about the abuse), but it’s nothing they can really do because they all way down there. That’s another reason why I was so worried about getting a place to stay.”

(1028)

“Since I’m alone here, and I don’t have family. Well, recently, one cousin is close, but I don’t have family here. For me, it was very important to stay in the place that I already knew to have some distant neighbors, at least, to ask for help. If I were in a totally new neighborhood, it would have been different.”

(1067)

Three women discussed the importance of family support and finding an appropriate distance between their home and those of their loved ones. Being within a short drive was prioritized over walking distance.

“I didn’t want to be too far away from my family, but I didn’t want to be so close that I can walk. (…) not super, super far to where it’s a 30-minute commute or anything like that.”

(1045)

Family provided relief from single parenting as well as moral support.

“Luckily, I do have family who would be willing to watch one or two of them, or my mom would take all three of them. (…) I just need somebody, some other adult supervision, to help. (laughs) She (mom) will take them off my hands and let me get some rest. It’s been pretty good.”

(1025)

My family, like my mom, my sisters, and my brothers, they’re all supportive of me. (…) They didn’t turn their backs on me because I took him back. (…) I still have all my family to stand behind me, like to go and talk to if I need to. They might not give me the advice I want to hear or sugarcoat things, but sometimes honesty and straightforwardness are what you really need.

(1045)

However, proximity to loved ones did not impact the housing selection of one who lacked family support. “Nobody never comes to see us. Nobody, no matter where I live.”

(1032)

#### 3.5.3. Housing Affordability Stress

Economic stressors led to survivors’ uncertainty about their ability to afford their new home. Survivors discussed the impact of unemployment and significant life events as additional financial setbacks regarding re-housing.

“I mean, my housing in itself is OK, but I live every day not knowing if I can pay the amount that needs to be paid because I’m not working.”

(1018)

“I’m just starting to pay my rent on my own. I had to get help from other sources because I got in an accident at work. The trailer hit me. I was out of work for a long time.”

(1032)

Financial challenges were exacerbated by reliance on limited public assistance.

“My current living situation, I live with myself and my two kids, my two sons. It’s not really stable because I lost my job when all of this stuff was going on (laughs). It’s a struggle trying to stay on a good foot. (…) Now I’m in debt because I’m struggling (after not being able to secure additional services).”

(1018)

Another survivor described how recent economic independence resulted in her ability to afford her basic needs only partially.

*“*I’m still a little behind and stuff. I’m not really sure if I’m going to be able to pay. I paid my rent for this month. I did do that, but I still have my BG&E [gas and electric] to pay. I still have my water to pay, my car insurance, and my car payment. Then it’s like, “Oh, my God. It’s so much stuff.” This is my very first house by myself. I’ve always lived with my mom, so I never really had to deal with all the bills being on top of me and having to stretch my money out, working paycheck to paycheck, and stuff like that. It’s a little scary because I really don’t know if I’m going to be able to. I’m going to try to stay positive. Don’t stress about tomorrow. Just worry about today. That’s all I really can do.”

(1045)

#### 3.5.4. Trouble Finding Housing

Long re-housing delays resulted from failed property inspections and administrative processes like a limited number of eligible inspectors.

*“*I filled out for this apartment about three months into being at the (shelter). I had to wait until I passed inspection. At first, I didn’t know what it was. I think he [the landlord] was just giving me the runaround. I applied, my finances were in order, everything was correct on my end. He kept telling me that we’re waiting to be scheduled. What happened is he got the inspection, and the inspection did not pass. Then, he kept having me waiting. Finally, the inspection came around again. That’s when I got it.”

(1031)

“I was supposed to move on April 5th, but it didn’t pass the inspection. Once they fixed whatever the inspector had told them, it took forever for the inspector to come back. So that pushed it all the way out. I think I moved in on May 30th.”

(1023)

“At first, when the inspectors came out, they had failed it twice, so I was getting a little discouraged and stuff because my counselor told me that the inspectors only go out three times, and if they deny it all three times, then I would have to look for somewhere else to go because they wasn’t going to keep coming out. Thankfully, the third time that the inspectors came out, everything was passed.”

(1045)

One survivor discussed perceived housing discrimination or feelings of stigma regarding her use of a housing voucher.

“You can get there, and some places make you feel as though you are beneath society because you need additional help from the government or from wherever you’re getting the help from. Sometimes, it makes you not want to visit certain places because you feel like they’re going to look at you like, oh, they want a handout.”

(1028)

Survivors also discussed being aware of the potential for predatory landlords who solicit application fees for unavailable properties.

“What was hard about it is what a lot of people don’t know or understand that it’s a lot of people that scam people out there. It didn’t happen to me per se. Because of the information that I got from someone else at the (shelter), I was able to not be in the same situation. What happened was a young lady went to go see a house. She put her money down on the house regarding an application fee. The person that was renting out the house did that with other people, meaning they collected all of the application fees, even though they knew that they already selected someone for that place. Eventually, when you do a lot of application fees, that’s almost a month’s rent, you know?”

(1010)

Survivors with unfavorable credit scores or low monthly income are particularly vulnerable to such scams. The same survivor shared how her financial standing and desperation landed her in a neighborhood where she felt unsafe.

“The trouble that I had also is even though I was going to have a voucher, they want you to have three times the income. (…) I had to tell them everything. Even if I was receiving money or something from someone as a gift, I had to put all of that down in order for me to get this place (new home) because a lot of the other places wasn’t working with me.”

(1010)

#### 3.5.5. Neighborhood Safety

Feeling desperate or rushed to find housing, limited housing stock, and delays may impact survivors’ housing choices and long-term housing stability—some survivors who felt rushed or desperate to find housing re-housed in neighborhoods where they felt unsafe.

“I felt like I made a poor choice as far as the neighborhood. I should have took time if I had more time, and I think that was the problem. I didn’t have enough time to really research the neighborhood.”

(1010)

“I don’t feel safe here no more. I’m ready to find a new house and move. It’s too much for me.”

(1048)

Survivors described neighborhood violence and damaged entry points (e.g., doors, windows) in their new home, which made them feel unsafe and limited social interaction. Neighborhood violence ranged from mail theft to homicide.

“I feel safe here besides them stealing my packages. That just makes me feel like I don’t want to live here no more. Then it makes me feel like somebody’s watching me, my every move, stuff like that.”

(1032)

“Since I moved in September, maybe about nine people have been killed in the neighborhood, and one across the street. It’s to the point that my kids really don’t go outside, and if they do, maybe the backyard. We pretty much stay inside the house, and I only l­­­eave out if I really have to.”

(1010)

“I don’t speak to nobody. I go to the store, buy me some cigarettes, and go back in the house.”

(1028)

One participant described having unrepaired maintenance issues that limited her safety.

“The front door has been kicked in before, and she (landlord) never fixed the frame. It still wobbles. The door’s still hard to close. The window doesn’t lock. It’s the front window. That where my daughter be right there playing her games. What if somebody comes through the window?”

## 4. Discussion

Housing determines one’s health and well-being. If we are keen to support IPV survivors achieve health and safety, we must be attentive to where they live and the impact of structural racism and gender discrimination. This study suggests that discriminatory housing practices and IPV may compromise survivors’ long-term health through neighborhood deprivation and residential segregation. Our findings capture the influence of economic dependence on an abusive partner, tradeoffs of safety for independent shelter due to socio-structural factors like housing stock and affordability, difficulty with landlords, and the lingering impacts of housing policies and practices such as redlining on the re-housing of recent IPV survivors. 

IPV survivors were re-housed in census tracts with significantly more neighborhood deprivation and segregation. Only 6% of survivors were able to meet their basic needs fully and independently, and one in ten were unable to meet their needs at all. Survivors’ limited resources resulted in food insecurity, economic barriers to re-housing, and dependence on their abusive partner. The characteristics of survivors in this study underscore the lingering economic impacts of IPV post-separation and competing priorities that survivors must navigate. 

Stigma about using housing vouchers, insufficient vouchers, and income qualifications are structural barriers that stifled housing selection in high-resource neighborhoods. Such established barriers strengthen concentrated deprivation [14]. Survivors who struggled to obtain housing in the past three months were significantly more likely to re-house in census tracts with a greater intensity of deprivation than those who did not experience trouble re-housing. It is possible that the former group attempted to re-house in neighborhoods with greater opportunity, though we did not measure this possible occurrence. Process barriers such as failed inspections delayed re-housing and may have influenced hasty decision-making leading to unsafe neighborhoods, as stated by survivors in this study, or re-housing in neighborhoods with greater deprivation. Further, IPV survivors may experience gender and class discrimination based on housing status and abuse experiences that prevent re-housing in less disadvantaged neighborhoods [26]. 

Findings may reflect the present-day manifestation of redlining and other forms of structural racism, where affordable housing is concentrated in areas of greatest deprivation and segregation. Most survivors in this sample earned less than $24,000 per year. Lack of income left many survivors stressed about their ability to afford housing. Such trepidation may have resulted in their conscious selection into more deprived neighborhoods based on housing affordability. According to our findings, survivors who were unable to meet their needs at all lived in significantly less deprived census tracts. These survivors may have prioritized safety or residence in an opportunity neighborhood over housing affordability. Conversely, survivors who could meet their needs independently or partially with assistance re-housed in census tracts with greater deprivation. These points are further supported by the impact of survivors’ perception of future IPV on housing selection. Survivors who were unsure of their risk of future abuse re-housed in significantly less deprived neighborhoods relative to those who did not fear future abuse. This trend was also observed in survivors who believed future abuse was likely (n = 5); they also re-housed in less deprived neighborhoods (n.s.). In other words, survivors who believed that future abuse was at least somewhat likely re-housed in less deprived neighborhoods with, perhaps, a greater perception of safety. These findings highlight the significant economic tradeoffs for safety and stability, where the prioritization of safety may exacerbate economic devastation for IPV survivors.

Malicious control of financial resources—economic abuse—is an understudied form of IPV associated with homelessness and housing instability among IPV survivors, as well as survivor dependency on the intimate partner [9,82]. Economic dependence on an abusive partner to meet basic needs like housing and children’s supplies were discussed by IPV survivors in the study. Dependence on the abusive partner predicted re-housing in significantly more deprived (unadjusted model) and segregated census tracts for reasons not discussed in interviews. Perhaps, the partners were unreliable in their support, a form of economic abuse [8], or lacked financial resources themselves. It may be possible that survivors re-housed in more deprived neighborhoods based on familiarity. Comfortability with the proximity to loved ones significantly predicted re-housing in a substantially more deprived neighborhood. This finding is consistent with the work of Jaramillo et al. (2020), where neighborhood safety and social cohesion were significantly associated with neighborhood satisfaction among housing voucher recipients [48]. Furthermore, gender- and race-wage gaps that marginalize women and people of color further exacerbate IPV-related housing insecurity and placement in deprived neighborhoods [83].

Several recommendations can be gleaned from this mixed-methods analysis. Research on safety planning among IPV survivors has shown tremendous benefit in empowering survivors’ safety decision-making [84,85]. Similarly, a decision aid specific to survivor re-housing could more formally facilitate identifying survivors’ housing priorities, establish neighborhood-level safety planning and assess neighborhood qualities as was suggested by one woman in the study. More generous housing subsidies to support re-housing transitions are paramount. Findings emphasize the significant economic challenges IPV survivors face in their pursuit of safety and stability. Current financial re-housing assistance seems insufficient in light of financial devastation brought on by IPV victimization (sabotaged credit and renting history, unemployment). Housing instability and homelessness are experienced across the life-course of IPV survivors. Additional economic support coupled with structural changes that facilitate housing mobility may decrease subsequent risk of IPV and housing instability among survivors [47].

Our study is the first to document the spatial distribution of housing selection among recently re-housed IPV survivors. However, findings should be considered in the context of the study’s limitations. First, our relatively small sample size, taken together with small cell sizes for certain variables, limits our analysis capabilities, including statistical power for multivariate modeling, though these models offer an important direction as to the nature, directionality, and potential effect sizes among variables. Further, the findings are inherently sensitive to the area-level unit chosen; we cannot observe variation within census tracts [86]. However, most residents live in census tracts surrounded by similar levels of deprivation and segregation [15]. Due to the racial/ethnic composition of RRH participants, we were unable to fully assess the effect of race/ethnicity or racism on housing selection. Some survivors were recruited for this study during the COVID-19 pandemic, which may bias our results. Post-hoc sensitivity analyses showed that recent difficulty with a landlord decreased between women enrolled before and after pandemic onset (*p* < 0.05). This shift may result from short-term housing protection implemented at the state and federal levels or the landlords’ desire to fill vacancies during a period of economic turmoil. However, NDI, residential segregation, and other key variables did not vary significantly between the two subgroups. It is also important to highlight that all women were recruited from a domestic violence shelter, thus representing the most economically deprived survivors. We see this as a strength, as women who leave an abusive partner have an increased homicide risk [12]. The generalizability of this study is unclear. However, this study takes place in the Baltimore-Washington Metropolitan Area, an area known for historic housing discrimination [49] and discrimination based on race and gender [87]. The stark divisions in neighborhood deprivation and residential segregation bolster the analysis. Our findings highlight the need for additional analyses of re-housing among IPV survivors.

## 5. Conclusions

Neighborhood deprivation and residential segregation represent a constellation of risk factors that may undermine IPV survivors’ safety and housing stability. Such factors result from racist policies that continue to be felt by IPV survivors based on their intersecting identities of race, ethnicity, and gender. Findings highlight the significant economic tradeoffs for safety and stability, such that survivors re-house in the most deprived and segregated neighborhoods within their respective regions. Neighborhood attributes should be considered in RRH programs for IPV survivors. Future studies should assess the long-term impacts of neighborhood risk factors on recently re-housed IPV survivors’ safety and stability.

## Figures and Tables

**Table 1:** Characteristics of female housing intervention participants, recent intimate partner violence (IPV) survivors, in the Baltimore-Washington Metropolitan Areas, *n* = 54.

	% (*n*)
DEMOGRAPHIC CHARACTERISTICS	
Age	
Less than 35 years	63 (34)
35 years or older	37 (20)
Race	
White	2 (1)
Black	76 (41)
Hispanic	7 (4)
Asian/Other	6 (3)
Multiracial	9 (5)
ECONOMIC CHARACTERISTICS	
Education	
Some HS	7 (4)
HS Graduate	35 (19)
At least some college	57 (31)
Income	
$0–$16,000	33 (17)
$16,001–$24,000	29 (15)
$24,001–$32,000	13 (7)
$32,001 or more	25 (13)
Past month employment (Yes)	65 (35)
Financial ability	
Meets needs independently	6 (3)
Meets needs with assistance	47 (25)
Partially meets needs with assistance	38 (20)
Cannot meet needs	9 (5)
Food stress	
Always or usually	40 (21)
Sometimes	28 (15)
Rarely/never	32 (17)
Economically dependent on partner	57 (31)
HOUSING CHARACTERISTICS	
Trouble getting housing, past 3 m	59 (29)
Recent difficulty with landlord	23 (12)
Housing affordability stress	
Always	59 (31)
Sometimes	28 (15)
Rarely/Never	13 (7)
HEALTH CHARACTERISTICS	
PTSD Symptoms	78 (42)
Unable to access necessary healthcare, past 12 m	17 (9)
Depression symptoms	39 (21)
SOCIAL/COMMUNITY CONTEXT	
Intimate partner violence, past 3 m	54 (28)
Feels safe at home	87 (47)
Co-parenting with abusive partner	70 (38)
Comfortable with proximity to family/friends	58 (31)
Comfortable with proximity to abuser	72 (38)

**Table 2:** Neighborhood deprivation index by individual, interpersonal, economic, and social factors among female housing intervention participants, recent IPV survivors, Baltimore, Maryland, and District of Columbia Metropolitan Areas, *n* = 54. Bold text signifies statistical significance.

		Neighborhood Deprivation Index Coeff., 95% CI		
	% (*n*)	M (SD)	Unadjusted	Adjusted
Range across four regions (847 census tracts)	-	-	−1.25, 4.30	-
Range across sample (43 census tracts)	-	-	−0.88, 3.53	-
Demographic Characteristics				
Age (ref: <35 years)	63 (34)	1.00 (1.09)	-	-
35 years or older	37 (20)	1.04 (1.24)	0.04 (−0.62, 0.71)	-
Race	-	-	0.20 (−0.55, 0.15)	-
Black	76 (41)	1.15 (1.13)	-	-
White	2 (1)	1.24 (.)	-	-
Hispanic	7 (4)	0.36 (0.88)	-	-
Asian/other	6 (3)	0.04 (1.04)	-	-
Multiracial	9 (5)	0.92 (1.42)	-	-
Economic Demographic Characteristics				
Education (Ref: At least some college)	57 (31)	0.91 (1.17)	-	-
Some HS	7 (4)	0.59 (1.24)	−0.32 (−1.51, 0.87)	-
HS Graduate	35 (19)	1.26 (1.08)	0.35 (−0.30, 1.00)	-
Income (Ref: $0–$16,000)	33 (17)	2.00 (1.17)	-	-
$16,001–$24,000	29 (15)	1.07 (1.14)	−0.13 (−0.96, 0.70)	−0.12 (−1.06, 0.81)
$24,001–$32,000	13 (7)	1.50 (1.26)	0.30 (−0.79, 1.39)	0.30 (−0.77, 1.37)
$32,001 or more	25 (13)	0.24 (0.51)	−0.96 (−1.61, −0.32) **	−0.46 (−1.30, 0.38)
Past month employment (Yes)	65 (35)	1.03 (1.21)	0.06 (−0.56, 0.68)	-
Financial Ability (Ref: Cannot meet needs)	9 (5)	0.04 (0.96)	-	-
Meets needs independently	6 (3)	1.16 (0.46)	1.12 (0.20, 2.03) **	0.88 (−0.87, 2.63)
Meets needs with assistance	47 (25)	0.99 (1.11)	0.94 (0.02, 1.86) **	0.82 (−0.70, 2.34)
Partially meets needs with assistance	38 (20)	1.15 (1.14)	1.10 (0.15, 2.06) **	0.81(−0.82, 2.44)
Economically dependent on partner	57 (31)	1.23 (1.29)	0.52 (−0.06, 1.10) *	0.31 (−0.25, 0.88)
Food stress (Ref: Always)	40 (21)	1.11 (1.35)	-	-
Sometimes	28 (15)	0.78 (0.90)	−0.33 (−1.08, 0.43)	-
Rarely/never	32 (17)	0.96 (0.95)	−0.15 (−0.91, 0.60)	-
Housing Characteristics				
Trouble getting housing, past 3 m	59 (29)	1.30 (1.28)	0.65 (0.04, 1.27) **	0.74 (0.13, 1.34) **
Recent difficulty with landlord	23 (12)	1.14 (1.57)	0.17 (−0.77, 1.11)	-
Housing affordability stress (Ref: Always)	59 (31)	1.00 (1.17)	-	-
Sometimes	28 (15)	0.78 (1.10)	−0.22(−0.93, 0.49)	-
Rarely/never	13 (7)	1.23 (0.79)	0.23 (−0.48, 0.95)	-
Social/Community Context				
Feels safe at home	87 (47)	1.07 (1.10)	0.45 (−0.61, 1.52)	-
Child(ren) with abusive partner	70 (38)	0.91 (1.11)	−0.33 (−1.03, 0.36)	-
Comfortable with proximity to family/friends	58 (31)	1.24 (1.00)	0.58 (−0.06, 1.22) *	0.75 (0.02, 1.48) **
Comfortable with proximity to abuser	72 (38)	1.12 (1.11)	0.44 (−0.26, 1.16)	-
Social cohesion (Ref: Neutral)	50 (27)	0.84 (0.99)	-	-
Yes	39 (21)	1.21 (1.32)	0.38 (−0.33, 1.078)	-
No	11 (6)	1.01 (1.09)	0.24 (−0.68, 1.16)	-
Intimate Partner Violence, past 3 m	54 (28)	1.12 (1.11)	0.23 (−0.42, 0.87)	-
Perceived risk of IPV, next 3 m (Ref: Unlikely)	69 (37)	1.20 (1.17)	-	-
Unsure	24 (13)	0.61 (0.91)	−0.59 (−1.22, 0.05) *	−0.76 (−1.39, −0.14) **
Likely	7 (4)	0.55 (1.30)	−0.65 (−1.84, 0.54)	−0.40 (−1.46, 0.66)
Constant	-	-	-	−0.59 (−2.30, 1.12)

* *p* < 0.10, ** *p* < 0.05.

**Table 3:** Residential segregation by individual, interpersonal, economic, and social factors among female housing intervention participants, recent IPV survivors, Baltimore, Maryland, and District of Columbia Metropolitan Areas, *n* = 54. Bold text signifies statistical significance.

		Residential Segregation Coeff., 95% CI		
	% (*n*)	M (SD)	Unadjusted	Adjusted
Range across four regions (847 census tracts)	-	-	0.99, −1	-
Range across sample (43 census tracts)	-	-	0.66, −1	-
Demographic Characteristics				
Age (ref: <35 years)	63 (34)	−0.53 (0.48)	-	-
35 years or older	37 (20)	−0.49 (0.51)	0.05 (−0.22, 0.33)	-
Race	-	-	-	-
Black	76 (41)	−0.59 (0.46)	-	-
White	2 (1)	−0.43 (.)	-	-
Hispanic	7 (4)	−0.09 (0.68)	-	-
Asian/other	6 (3)	−0.27 (0.25)	-	-
Multiracial	9 (5)	−0.42 (0.57)	-	-
Economic Demographic Characteristics				
Education (Ref: At least some college)	57 (31)	−0.43 (0.48)	-	-
Some HS	7 (4)	−0.73 (0.30)	−0.30 (−0.62, 0.02) *	−0.49 (−0.88, −0.09) **
HS Graduate	35 (19)	−0.61 (0.51)	−0.19 (−0.48, 0.10)	−0.26 (−0.57, 0.04) *
Income (Ref: $0–$16,000)	33 (17)	−0.44 (0.62)	-	-
$16,001–$24,000	29 (15)	−0.47 (0.48)	−0.04 (−0.42, 0.36)	0.01 (−0.36, 0.37)
$24,001–$32,000	13 (7)	−0.80 (0.14)	−0.37 (−0.69, 0.04) **	−0.32 (−0.67, 0.02) *
$32,001 or more	25 (13)	−0.48 (0.41)	−0.05 (−0.43, 0.33)	−0.09 (−0.49, 0.31)
Past month employment (Yes)	65 (35)	−0.52 (0.45)	−0.01 (−0.30, 0.29)	
Financial Ability (Ref: Cannot meet needs)	9 (5)	−0.39 (0.47)		-
Meets needs independently	6 (3)	−0.82 (0.11)	−0.43 (−0.83, −0.02) **	−0.46 (−1.16, 0.25)
Meets needs with assistance	47 (25)	−0.46 (0.53)	−0.07 (−0.52, 0.38’)	−0.22 (−0.72, 0.27)
Partially meets needs with assistance	38 (20)	−0.55 (0.46)	−0.15 (−0.60, 0.29)	−0.31 (−0.80, 0.19)
Economically dependent on partner	57 (31)	−0.61 (0.43)	−0.23 (−0.50, 0.03) *	−0.31 (−0.56, −0.06) **
Food stress (Ref: Always)	40 (21)	−0.60 (0.41)		-
Sometimes	28 (15)	−0.36 (0.58)	0.24 (−0.11, 0.59)	-
Rarely/never	32 (17)	−0.52 (0.47)	0.08 (−0.21, 0.37)	-
Housing Characteristics				
Trouble getting housing, past 3 m	59 (29)	−0.53 (0.52)	−0.05 (−0.33, 0.23)	-
Recent difficulty with landlord	23 (12)	−0.59 (0.32)	−0.11 (−0.36, 0.13)	-
Housing affordability stress (Ref: Always)	59 (31)	−0.57 (0.44)	-	-
Sometimes	28 (15)	−0.28 (0.57)	0.29 (−0.05, 0.63) *	0.39 (0.04, 0.75) **
Rarely/never	13 (7)	−0.69 (0.32)	−0.12 (−0.40, 0.17)	−0.07 (−0.47, 0.31)
Social/Community Context				
Feels safe at home	87 (47)	−0.54 (0.46)	−0.21 (−0.67, 0.24)	-
Child(ren) with abusive partner	70 (38)	−0.51 (0.49)	0.02 (−0.27, 0.31)	-
Comfortable with proximity to family/friends	58 (31)	−0.60 (0.45)	−0.23 (−0.49, 0.05)	-
Comfortable with proximity to abuser	72 (38)	−0.55 (0.42)	−0.15 (−0.50, 0.20)	-
Social cohesion (Ref: Neutral)	50 (27)	0.52 (0.52)	-	-
Yes	39 (21)	−0.48 (0.44)	0.05 (−0.23, 0.33)	-
No	11 (6)	−0.61 (0.52)	−0.08 (−0.53, 0.37)	-
Intimate Partner Violence, past 3 m	54 (28)	−0.55 (0.45)	−0.11 (−0.38, 0.17)	-
Perceived risk of IPV, next 3 m (Ref: Unlikely)	69 (37)	−0.57 (0.43)	-	-
Unsure	24 (13)	0.37 (0.63)	0.20 (−0.18, 0.57)	-
Likely	7 (4)	−0.46 (0.39)	0.11 (−0.27, 0.49)	-
Constant	-	-	-	0.01 (−0.60, 0.62)

* *p* < 0.10, ** *p* < 0.05.

## Data Availability

Restrictions apply to the availability of these data. Data may be made available with the permission of the senior author, M.R.D.
